# The Efficacy of Sodium Bisulfate Salt (SBS) Alone and Combined With Peracetic Acid (PAA) as an Antimicrobial on Whole Chicken Drumsticks Artificially Inoculated With *Salmonella* Enteritidis

**DOI:** 10.3389/fvets.2019.00006

**Published:** 2019-01-30

**Authors:** Dana K. Dittoe, Julie A. Atchley, Kristina M. Feye, Jung Ae Lee, Carl J. Knueven, Steven C. Ricke

**Affiliations:** ^1^Department of Food Science and Center for Food Safety, University of Arkansas, Fayetteville, AR, United States; ^2^Agricultural Statistics Laboratory, University of Arkansas, Fayetteville, AR, United States; ^3^Jones-Hamilton Co., Walbridge, OH, United States

**Keywords:** *S*. Enteritidis, sodium bisulfate salt, processing, poultry, part dips

## Abstract

The presence of *Salmonella* spp. on poultry products is one of the leading causes of foodborne illness in the United States. Therefore, novel antimicrobial substances are being explored as potential interventions in poultry processing facilities. The objective of the current study was to evaluate the efficacy of varying concentrations of sodium bisulfate salt, SBS, alone or in combination with peracetic acid, PAA, in 15 s whole part dips. Ninety six drumsticks (4 replications, 8 treatments, 3 days) were inoculated separately in a 400 mL solution of nalidixic resistant (NA) *Salmonella* Enteritidis (10^7^ CFU/mL) and allowed to adhere for 60 to 90 min at 4°C for a final concentration of 10^6^ CFU/g. The experimental treatments included: a no treatment (control), and 15 s dips in 300 mL of tap water alone (TW) or with the addition of 1; 2; and 3% SBS; 1; 2; and 3% SBS+PAA. After treatment, drumsticks were stored at 4°C until microbial sampling was conducted. On d 0, l, and 3, drumsticks were rinsed in 150 mL of nBPW for 1 min, 100 μL of rinsate was serially diluted, spread plated on XLT4+NA (20 μg/mL), and incubated aerobically at 37°C for 24 h. Log-transformed counts were analyzed using a randomized complete block design (day) using One-Way ANOVA, polynomial contrasts, and pairwise comparisons with means being separated by Tukey's HSD with a significance level of *P* ≤ 0.05. A treatment by day interaction (*P* = 0.14071) was not substantial. Thus, the treatment effect was investigated separately by days. Over time, a linear trend was observed in *S*. Enteritidis concentration when SBS was increased (1 < 2 < 3%). The concentration of *S*. Enteritidis was different between 1% SBS and 1% SBS+PAA on d 0. However, the level of *S*. Enteritidis was not different among drumsticks treated in 2 and 3% SBS and 2 and 3% SBS+PAA across d 0, 1, 3. The application of 3% SBS alone or in combination with 200 ppm of PAA is capable of reducing the presence of *Salmonella* over a 3-d refrigeration period; potentially increasing the safety of poultry products for consumers.

## Introduction

There is a need to enhance food safety strategies in the poultry industry. In the past 25 years, numerous steps have been taken to lessen the contamination of poultry products. The implantation of the Hazzard Analysis Critical Control Point (HACCP) in 1996 (1) and Food Safety Modernization Act (FSMA) in 2011 (2) established rules and guidelines for the food industry to follow in order to reduce the intensity and amount of foodborne illnesses. Although these strategies have had an impact on the incidence of foodborne illness, they are not entirely sufficient alone. As a result, the Center for Disease Control and Prevention (CDC) has set limits and goals on microbial reduction in the poultry industry. One pathogen the CDC is particularly interested in is *Salmonella*, especially Enteritidis, with outbreaks linked to poultry and eggs (3, 4). The CDC aims to lower *Salmonella* incidence by 5% by the year 2020 (5). In order to meet the CDC's goal, further intervention strategies must be integrated into the poultry industry.

In poultry processing facilities in the United States, one of the most commonly used methods to decontaminate poultry meat are antimicrobial washes and sprays at various locations during processing (6). Traditionally, chlorine and peracetic acid (PAA) are the antimicrobials of choice in the chiller, post chiller, spray cabinets, and part dips in poultry processing facilities (6). Recently, alternative antimicrobials have emerged for industrial application such as organic acids, phosphates, chlorine derivatives, and hydrogen peroxide solutions (7, 8). Antimicrobials that lower the pH of the surrounding environment are promising; however, Gram-negative species, such as *Salmonella*, are capable of developing resistance to organic acids due to the presence of LPS (9). In addition, bacteria such as *Salmonella* can build a tolerance to stressful environments (10).

Inorganic acids also have the potential to induce the resistance of pathogens, such as *Salmonella*, to a low environmental pH (11). Research conducted by Foster and Hall (11) revealed that in order for inorganic acids to induce an acid tolerance response new protein synthesis and the development of a pH homeostasis system is required. As a consequence, both the acid tolerance response and acid-shock proteins are required for *Salmonella* to survive acidic conditions induced by inorganic acids (12–14). Sodium bisulfate (SBS), an inorganic acid, has demonstrated the ability to decrease the extracellular pH to around 2 with a pK_a_ of 1.9 (15). If there is a mild decrease in the extracellular pH, *Salmonella* reduce cytoplasmic pH to maintain a neutral state (16). This response is incredibly arduous for *Salmonella* and can lead to cell death (16). Because of these features, SBS demonstrates the potential to be a valid antimicrobial over other organic and inorganic acids as it has the ability to create a highly acidic environment that is not easily adapted to.

Historically, SBS has been utilized commercially as an acidifier on poultry litter. When used as a litter amendment at high doses, SBS not only reduced ammonia volatilization from poultry litter but also the presence of *Salmonella* (17). Additionally, the dietary inclusion of SBS decreased the shedding of *Salmonella* into the litter (18). Pertinent to poultry processing, SBS was used as an antimicrobial rinse agent on apples to reduce artificially inoculated *Listeria monocytogenes* (19). The Environmental Protection Agency has declared SBS as a safer choice as an antimicrobial and processing aid (20). In addition, according to the World Health Organization (WHO), the use of SBS is approved with no restrictions on allowable daily intake in over 150 countries that recognize the WHO codex (21). Because of all of the preliminary data as well as the Environmental Protection Agency designation, it is evident that SBS could be a valid agent for reducing *Salmonella*, during multiple stages of poultry production, including processing. Therefore, it was the objective of the current study to investigate the potential of SBS as an antimicrobial intervention in poultry processing by determining the efficacy of SBS alone (1, 2, and 3%) or in combination with PAA (200 ppm) on mitigating the presence of a nalidixic acid (NA) resistant strains of *Salmonella* Enteritidis on whole chicken drumsticks.

## Materials and Methods

### Whole Chicken Drumsticks and *Salmonella* Screening

A total of 96 drumsticks (8 treatments, 3 days, 4 replicates) were obtained from a local supermarket no longer than 24 h before the onset of the study and chosen based on the furthest expiration date. Prior to the start of the study, one drumstick was screened for the background unintended presence of *Salmonella*. One drumstick was rinsed in 150 mL of neutralizing Buffered Peptone Water [nBPW; (22)] and manually agitated for 1 min. Subsequently, 100 μL of the rinsate was spread plated onto Xylose Lysine Tergitol 4 with the addition of 20 μg/mL of NA (XLT 4 + NA) and incubated for 24 h at 37°C.

### Inocula Preparation

Before the onset of the current study, a frozen stock of *Salmonella* Enteritidis that was selected to be resistant to 20 μg/mL of NA was streaked for isolation on Tryptic Soy Agar (TSA) and incubated for 24 h aerobically at 37°C. Subsequently, one isolated colony from the incubated plate was streaked onto fresh TSA with the addition of 20 μg/mL of NA (TSA + NA) and incubated under the previously mentioned conditions. Simultaneously, an isolated colony was streaked onto XLT 4 plus 20 μg/mL of NA (XLT 4 + NA) for confirmation and incubated aerobically at 37°C for 24 h. An isolated colony from the incubated TSA + NA plate was then transferred to 40 mL of fresh Tryptic Soy Broth the addition of 20 μg/mL of NA (TSB +NA) and incubated aerobically at 37°C in a shaking incubator at 200 rpm for 12 to 16 h. The resulting cultures of *S*. Enteritidis were determined to contain 10^8^ CFU/mL.

Directly following the overnight (12 to 16 h) incubation of the *S*. Enteritidis cultures, the cultures were spun down at 18,000 g for 5 min, decanted, followed by washing twice in 1 × Phosphate Buffered Saline (PBS). After the final wash, the pellet was resuspended in 400 mL of sterile DI water.

### Inoculation

A separate inoculum of *S*. Enteritidis was utilized per replication of drumsticks (4 replications). Approximately 24 drumsticks were placed into sterile Whirl-Pak® (Nasco, Atkins, WI, USA) bags, where the inoculum was administered. The inoculated drumsticks were then massaged manually for 5 min and allotted 60 to 90 min at 4°C to allow for attachment.

### Treatment pH and Application

Following inoculation, the eight experimental antimicrobial treatments were created by combining TW with the appropriate amounts of SBS and 200 ppm of PAA (Sigma-Aldrich, St. Louis, MO, USA) to create proper concentration of the following treatments: a no treatment (control), a 15 s dip in 300 mL of tap water alone (TW), and TW with the addition of 1% SBS (TW+SBS1); 2% SBS (TW+SBS2); 3% SBS (TW+SBS3); 1% SBS + PAA (TW+SBS1+PAA); 2% SBS + PAA (TW+SBS2+PAA); and 3% SBS + PAA (TW+SBS3+PAA). Before drumsticks were treated, one replicate of each treatment was analyzed for pH with a SympHony pH meter and probe (VWR International, Radnor, PA, USA). Immediately following the attachment period, the drumstick weights were recorded and the treatments were administered. The whole chicken drumsticks were independently dipped for 15 s into sterile Whirl-Pak bags containing the eight previously described treatments.

### Microbial Analysis and *Salmonella* Enumeration

Following the 15 s dips, the drumsticks were transferred to new sterile Whirl-Pak bags and allowed to rest for 2 min. The drumsticks were evaluated immediately on d 0 or maintained at 4°C for an additional 24 h (d 1) or 72 h (d 3) and then analyzed for *Salmonella* Enteritidis concentration. At each time point post-treatment, d 0, 1, and 3, the drumsticks were rinsed with 150 mL of sterile nBPW. The Whirl-Pak bags containing the 150 mL of nBPW and drumsticks were then manually agitated for 1 min and the resulting rinsates were collected for downstream analysis.

Whole chicken drumstick rinsates were aliquoted to 1.5 mL microcentrifuge tubes and subsequently serially diluted to 10^−6^ (1:10 dilution factor). After diluting the samples, a 100 μL aliquot of each dilution was spread plated onto XLT 4 + NA (20 μg/mL) agar in duplicate using sterile spreaders. The plates were then inverted and incubated aerobically for 24 h at 37°C. Only the plated dilutions with CFU counts between 30 and 300 were enumerated and recorded.

The following equation was utilized to calculate the CFU of *Salmonella* per gram of whole chicken drumstick:

$$\begin{array}{l} \frac{\left(\frac{Number\;of\;colonies}{0.1\;mL\;plated}\right)*\text{Dilution Factor}}{\frac{\text{Drumstick Weight}\left(\text{g}\right)}{\text{Original Homogenate}\left(\text{mL}\right)}}=\text{CFU}/\text{gram of Drumstick} \end{array}$$

### Statistical Analysis

Each drumstick was randomly assigned to a treatment and a time point prior to analyses. The CFU of *Salmonella* were log_10_ transformed and reported on a log CFU of *S*. Enteritidis per gram of drumstick basis (log CFU/g). The data were analyzed as a Randomized Complete Block design with replications (*n* = 4) where the blocks are designated as day, d 0, 1, and 3, using one-way ANOVA, polynomial contrasts, and pairwise comparisons. The differences were assessed statistically by using Tukey's protected HSD at 0.05 level of significance. Data analyses were performed in R version 3.3.2 (23).

## Results

**Figure 1:**
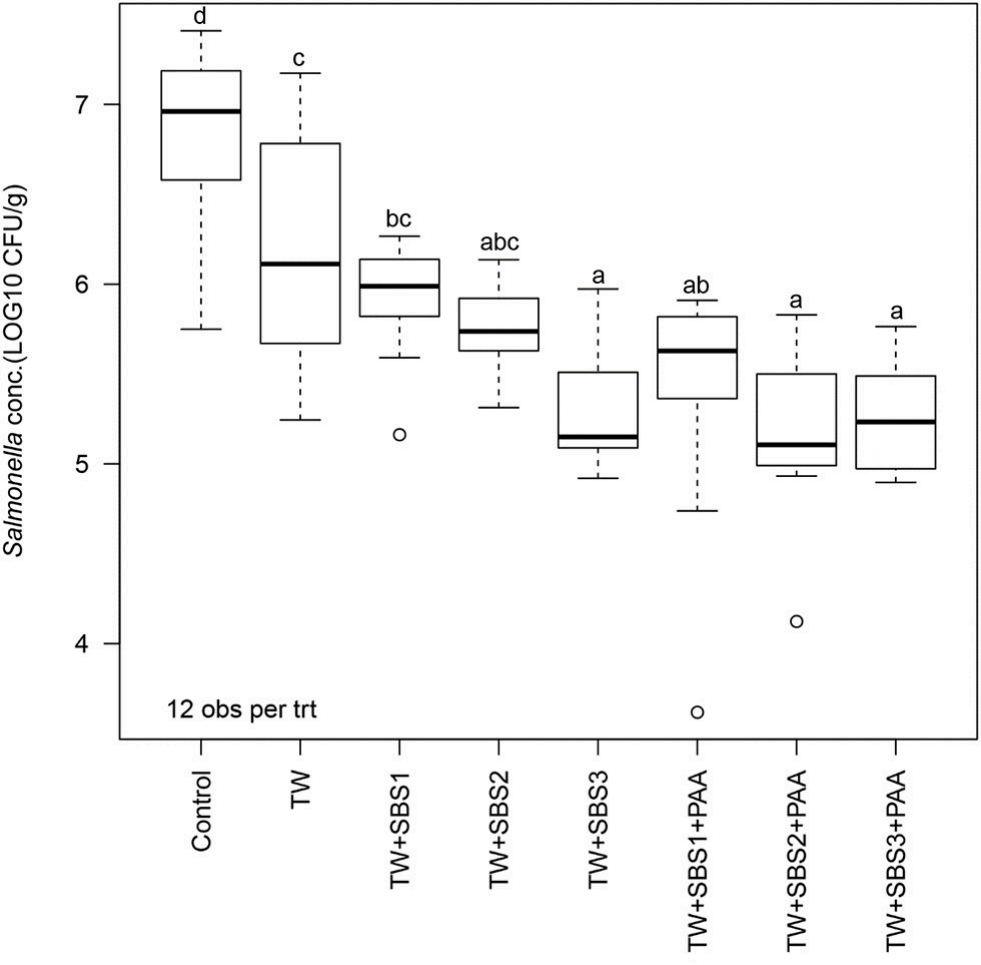
The effect of Sodium Bisulfate, SBS, and 200 ppm of peracetic acid, PAA, utilized alone or in combination as an antimicrobial 15 s part dip on the population of *Salmonella* Enteritidis on whole chicken drumsticks. In the current study, drumsticks were artificially inoculated with 10^7^ CFU/g of *S*. Enteritidis and subsequently treated in 300 mL of antimicrobial treatments to identify the remaining population of *Salmonella*. There were eight treatments consisting of: a no treatment Control, tap water (TW), tap water with the addition of either 1, 2, or 3% SBS indicated as TW+SBS1, TW+SBS2, and TW+SBS3, and the combination of 1, 2, and 3% SBS with 200 ppm of peracetic acid (PAA), represented as TW+SBS1+PAA, TW+SBS2+PAA, and TW+SBS3+PAA. The current figure demonstrates the effect the treatments had on *Salmonella* population regardless of refrigeration (4°C) time, d 0, 1, and 3. Individual standard error of the mean (SEM) for Control, TW, TW+SBS1, TW+SBS2, TW+SBS3, TW+SBS1+PAA, TW+SBS2+PAA, and TW+SBS3+PAA were 0.136, 0.180, 0.089, 0.066, 0.091, 0.190, 0.132, 0.087 log CFU/g, respectively. *F*-test *P*-value < 0.0001; Pooled SEM = 0.129; *N* = 96; *n* = 12. Means with different superscripts are considered different (a–d).

In the current study, the overall one-way ANOVA did not produce a significant interaction between the block (day) and treatment (*P* = 0.1407). There was no main effect of day (*P* = 0.0948); however, there was a main effect of treatment (*P* < 0.0001; Figure 1). Overall, all treatments, TW, TW+SBS1, TW+SBS2, TW+SBS3, TW+SBS1+PAA, TW+SBS2+PAA, and TW+SBS3+PAA (6.22, 5.93, 5.76, 5.28, 5.42, 5.19, 5.27 log CFU/g), reduced the population of *S*. Enteritidis on the drumsticks compared to the no treatment control (6.85 log CFU/g). Also, drumsticks treated with TW+SBS3, TW+SBS2+PAA, and TW+SBS3+PAA (5.28, 5.19, and 5.27 log CFU/g) had populations of *S*. Enteritidis 1 to 2 log CFU per g of drumstick lower than those treated with the Control, TW, and TW+SBS1 (6.85, 6.22, and 5.93 log CFU/g).

**Figure 2:**
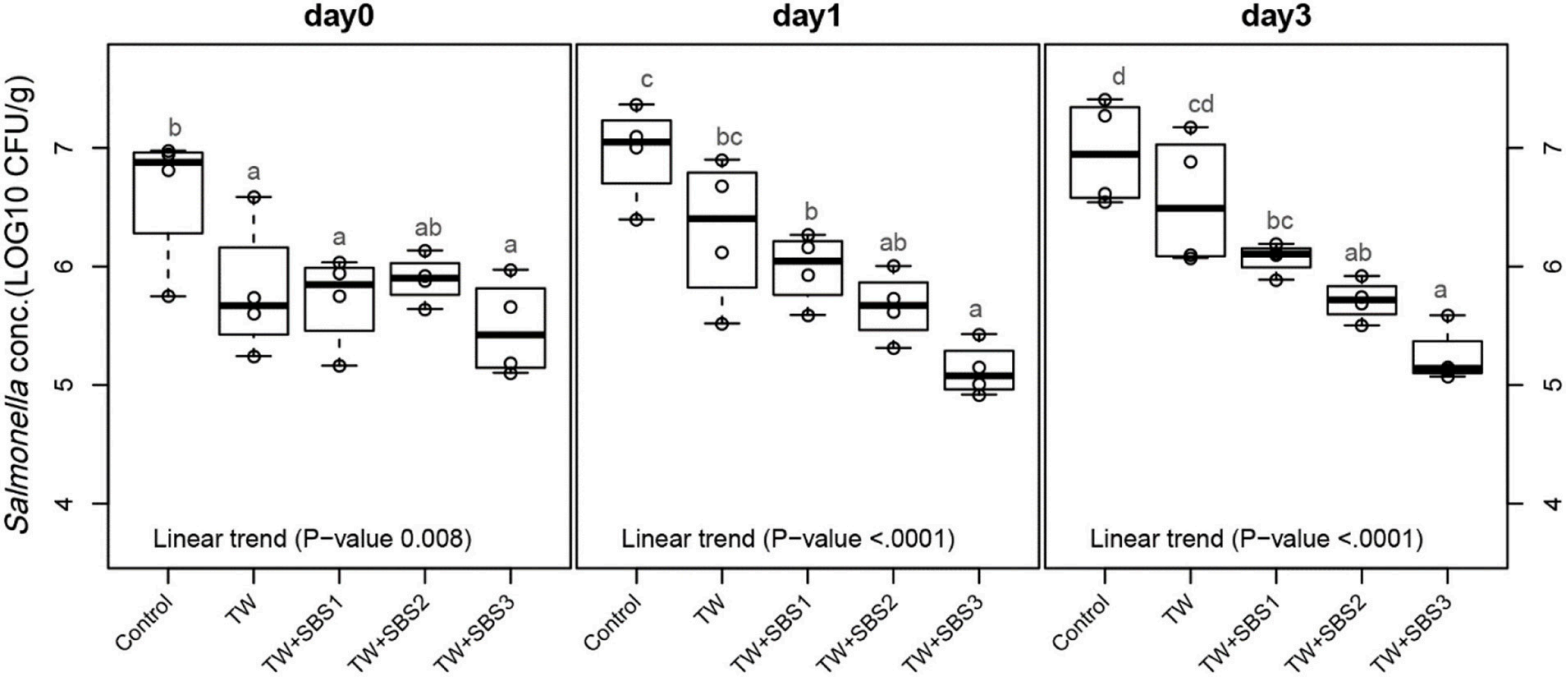
The linear effect of Sodium Bisulfate, SBS, as an antimicrobial 15 s part dip on suppressing the population of *Salmonella* Enteritidis on whole chicken drumsticks on d 0, 1, and 3. Drumsticks were artificially inoculated with 10^7^ CFU/g of *S*. Enteritidis and subsequently treated in 300 mL of antimicrobial treatments to identify the remaining population of *Salmonella*. There were eight antimicrobial treatments consisting of: a no treatment Control, tap water (TW), tap water with the addition of either 1, 2, or 3% SBS indicated as TW+SBS1, TW+SBS2, and TW+SBS3, and the combination of 1, 2, and 3% SBS with 200 ppm of peracetic acid (PAA), represented as TW+SBS1+PAA, TW+SBS2+PAA, and TW+SBS3+PAA. In the current figure, a linear trend was investigated for the incremental increase in SBS concentration, Control (*n* = 4), TW (*n* = 4), TW+SBS1 (TW+SBS1 and TW+SBS1+PAA, *n* = 8), TW+SBS2 (TW+SBS2 and TW+SBS2+PAA, *n* = 8), and TW+SBS3 (TW+SBS3 and TW+SBS3+PAA, *n* = 8), over a 3-d refrigeration period at 4°C. Individual SEM for Control, TW, TW+SBS1, TW+SBS2, and TW+SBS3 was 0.292, 0.284, 0.281, 0.229, 0.146 log CFU/g for d 0, 0.205, 0.309, 0.092, 0.122, and 0.089 log CFU/g for d 1, and 0.223, 0.278, 0.082, 0.131, and 0.067 for d 3, respectively. Pooled SEM for d 0 is 0.227, 0.196 for d 1, and 0.175 for d 3; *N* = 32. Means with different superscripts are considered different (a–d).

Although there was not a significant interaction between treatment and day (*P* = 0.1407), the main effects of each treatment on each day were evaluated using polynomial contrasts and pairwise comparisons of treatments. Linear trends were investigated for the increasing concentrations of SBS within treatments: Control, TW, TW+SBS1 (SBS1 and SBS1+PAA), TW+SBS2 (SBS2 and SBS2+PAA), and TW+SBS3 (SBS3 and SBS3+PAA). By combining treatments with similar concentrations of SBS, negative linear trends of log CFU of *Salmonella* per gram of drumstick occurred as SBS increased (1% < 2% < 3%) on d 0, 1, and 3 (*P* = 0.0008, *P* < 0.0001, *P* < 0.0001, Figure 2), where d 1 and 3 had distinct linear trends. On d 0, there was no detectable difference between TW+SBS 1, 2, and 3; however, on both d 1 and 3, TW+SBS1 and TW+SBS3 had detectable differences with SBS3 yielding a lower population of *S*. Enteritidis per gram of drumstick (5.99 and 5.13 log CFU/g on d 1 and 6.07 and 5.24 log CFU/g on d 3).

**Figure 3:**
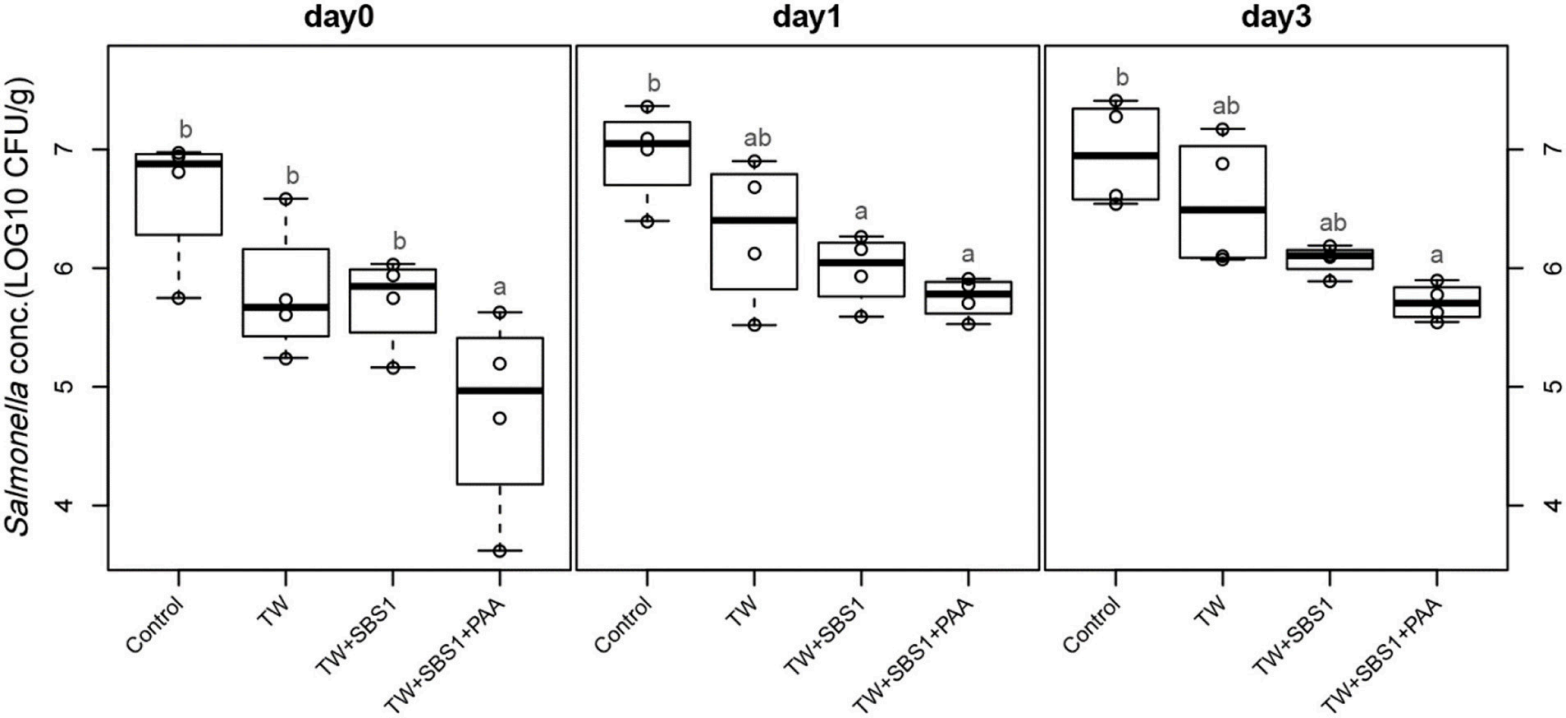
The comparative effect of 1% Sodium Bisulfate, SBS, and 200 ppm of peracetic acid, PAA, utilized alone or in combination as antimicrobial 15 s part dips on the presence of *Salmonella* Enteritidis on whole chicken drumsticks on d 0, 1, and 3. In the current study, drumsticks were artificially inoculated with 10^7^ CFU/g of *S*. Enteritidis and subsequently treated in 300 mL of antimicrobial treatments to identify the remaining population of *Salmonella*. In the study, there were eight treatments, consisting of: a no treatment Control, tap water (TW), tap water with the addition of either 1, 2, or 3% SBS indicated as TW+SBS1, TW+SBS2, and TW+SBS3, and the combination of 1, 2, and 3% SBS with 200 ppm of peracetic acid (PAA), represented as TW+SBS1+PAA, TW+SBS2+PAA, and TW+SBS3+PAA. However, in the current figure only the Control, TW, TW+SBS1, and TW+SBS1+PAA is represented and is separated by d 0, 1, and 3 of 4°C incubation. Individual SEM for Control, TW, TW+SBS1, and TW+SBS1+PAA was 0.293, 0.284, 0.196, and 0.432 for d 0; 0.205, 0.309, 0.150, and 0.086 for d 1; and 0.223, 0.278, 0.064, and 0.078 for d 3, respectively. *P*-value for d 0 is 0.0116, 0.0067 for d 1, and 0.0024 for d 3; Pooled SEM for d 0 is 0.313, 0.204 for d 1, and 0.0024 for d 3; Per day *N* = 16 and *n* = 4. Means with different superscripts are considered different (a,b).

**Figure 4:**
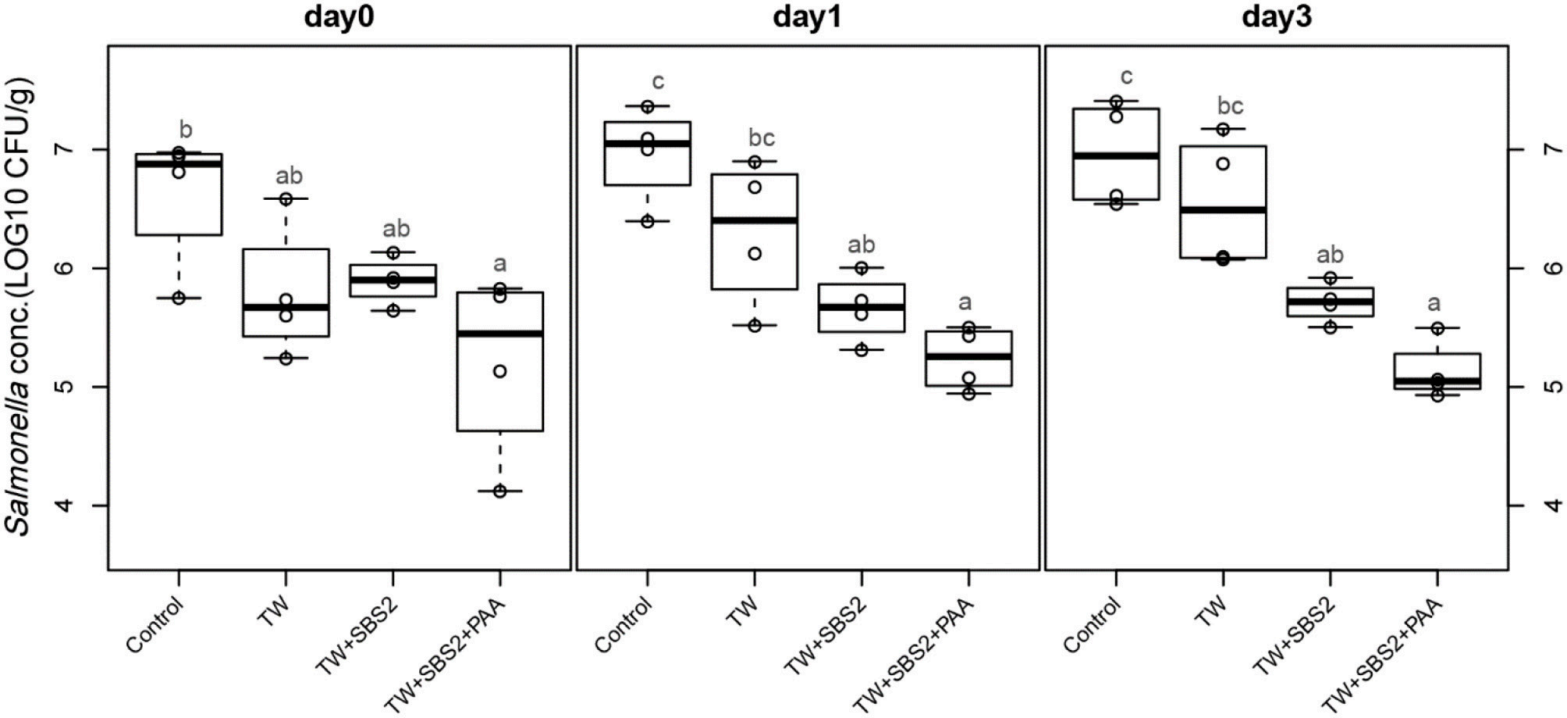
The comparative effect of 2% Sodium Bisulfate, SBS, and 200 ppm of peracetic acid, PAA, utilized alone or in combination as antimicrobial 15 s part dips on the population of *Salmonella* Enteritidis on whole chicken drumsticks on d 0, 1, and 3. In the current study, drumsticks were artificially inoculated with 10^7^ CFU/g of *S*. Enteritidis and subsequently treated in 300 mL of antimicrobial treatments to identify the remaining population of *Salmonella*. In the study, there were eight treatments, consisting of: a no treatment Control, tap water (TW), tap water with the addition of either 1, 2, or 3% SBS indicated as TW+SBS1, TW+SBS2, and TW+SBS3, and the combination of 1, 2, and 3% SBS with 200 ppm of peracetic acid (PAA), represented as TW+SBS1+PAA, TW+SBS2+PAA, and TW+SBS3+PAA. However, in the current figure only the Control, TW, TW+SBS2, and TW+SBS2+PAA is represented and is separated by d 0, 1, and 3 of 4°C incubation. Individual SEM for Control, TW, TW+SBS2, and TW+SBS2+PAA was 0.293, 0.284, 0.101, and 0.396 for d 0; 0.205, 0.309, 0.143, and 0.136 for d 1; and 0.223, 0.278, 0.086, and 0.125 for d 3, respectively. *P*-value for d 0 is 0.0343, 0.0005 for d 1, and < 0.0001 for d 3; Pooled SEM for d 0 is 0.289, 0.210 for d 1, and 0.194 for d 3; Per day *N* = 16 and *n* = 4. Means with different superscripts are considered different (a,c).

**Figure 5:**
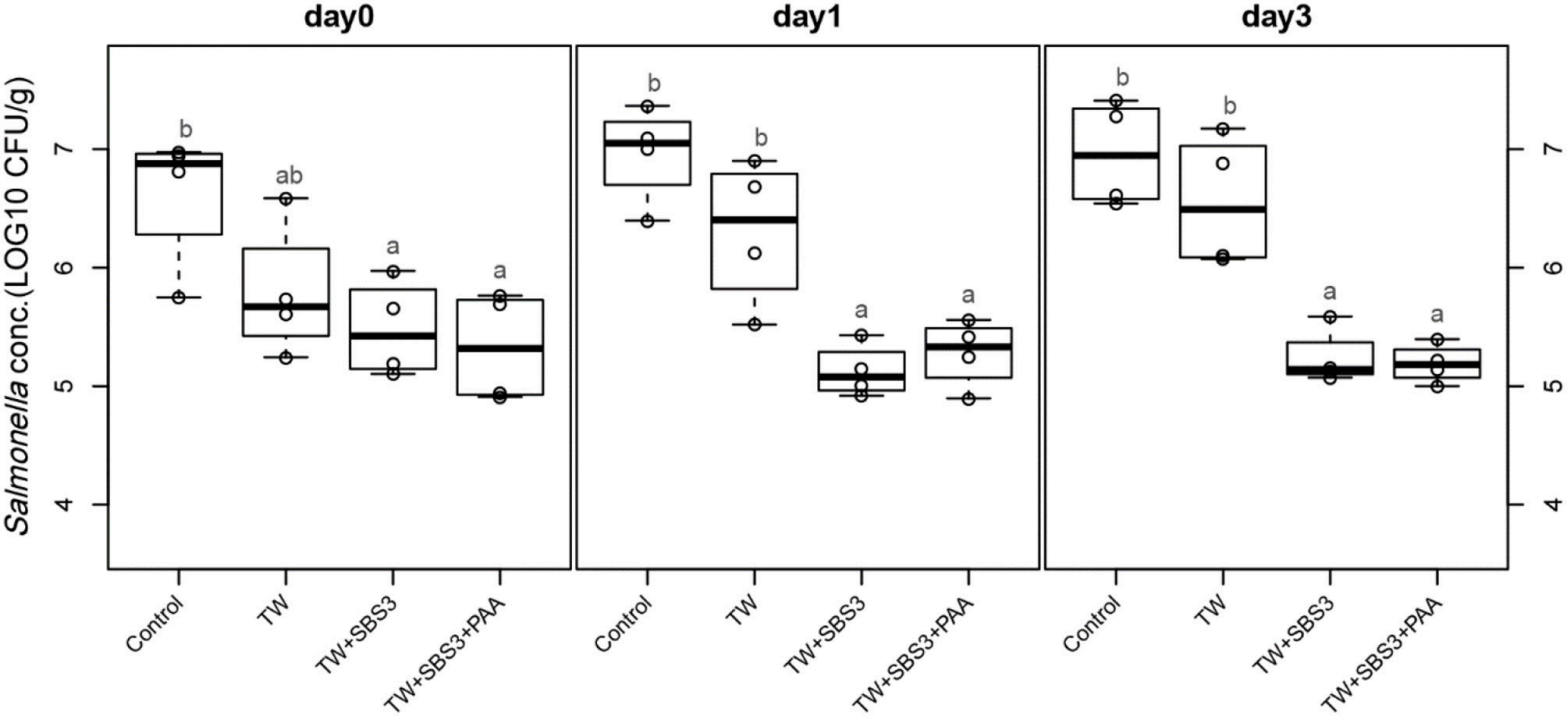
The comparative effect of 3% Sodium Bisulfate, SBS, and 200 ppm of peracetic acid, PAA, utilized alone or in combination as antimicrobial 15 s part dips on the presence of *Salmonella* Enteritidis on whole chicken drumsticks on d 0, 1, and 3. In the current study, drumsticks were artificially inoculated with 10^7^ CFU/g of *S*. Enteritidis and subsequently treated in 300 mL of antimicrobial treatments to identify the remaining population of *Salmonella*. In the study, there were eight treatments, consisting of: a no treatment Control, tap water (TW), tap water with the addition of either 1, 2, or 3% sodium bisulfate (SBS) indicated as TW+SBS1, TW+SBS2, and TW+SBS3, and the combination of 1, 2, and 3% SBS with 200 ppm of peracetic acid (PAA), represented as TW+SBS1+PAA, TW+SBS2+PAA, and TW+SBS3+PAA. However, in the current figure only the Control, TW, TW+SBS3, and TW+SBS3+PAA is represented and is separated by d 0, 1, and 3 of 4°C incubation. Individual SEM for Control, TW, TW+SBS3, and TW+SBS3+PAA was 0.293, 0.284, 0.205, and 0.232 for d 0; 0.205, 0.309, 0.112, and 0.143 for d 1; and 0.223, 0.278, 0.119, and 0.083 for d 3, respectively. *F*-test *P*-values are 0.0169 for d 0, 0.0001 for d 1, and < 0.0001 for d 3; Pooled SEMs are 0.256 for d 0, 0.206 for d 1, and 0.192 for d 3; Per day *N* = 16 and *n* = 4. Means with different superscripts are considered different (a,b).

To further evaluate the effects of the treatments, TW+SBS and TW+SBS+PAA treatments were examined separately alongside the control and TW in pairwise comparisons by day (Figures 3–5). Although not statistically different, TW+SBS1+PAA and TW+SBS2+PAA treatments exhibited a lower presence of *S*. Enteritidis per gram of drumstick than those treated with TW+SBS1 and TW+SBS2 alone. Only on d 0 did TW+SBS1+PAA have a significantly lower population of *S*. Enteritidis than TW+SBS1 (4.80 and 5.72 log CFU/g). Thus, both TW+SBS1+PAA and TW+SBS2+PAA treatments show a slight advantage over TW+SBS treatments. Drumsticks treated with TW+SBS3 and TW+SBS3+PAA did not yield the previously mentioned pattern. In fact, TW+SBS3+PAA (5.33, 5.28, and 5.19 log CFU/g on d 0, 1, and 3, respectively) was not more effective at reducing the population of *S*. Enteritidis on drumsticks than TW+SBS3 (5.48, 5.13, and 5.24 log CFU/g on d 0, 1, and 3, respectively), primarily as time continued.

Though the pH of the treatments was not statistically analyzed, due to insufficient replication, there was a clear numerical difference between TW and treatments (Table 1). The mean pH of the TW solution was 7.42; whereas, the mean pH of TW+SBS1, TW+SBS2, TW+SBS3, TW+SBS1+PAA, TW+SBS2+PAA, and TW+SBS3+PAA was 1.64, 1.45, 1.31, 1.51, 1.33, and 1.29, respectively. It should be noted that there was a numerical drop in the pH level of the TW+SBS1 and TW+SBS2 solutions when PAA was added. There was not a substantial drop in TW+SBS3 when PAA was added.

**Table 1:** The mean pH values of Sodium Bisulfate (SBS) salt and PAA, used alone or in combination when utilized as 15 s dip solutions^a^.

**Treatment^b^**	**Mean pH of Part Dips^c^**
Control	–
TW	7.42
TW + SBS1	1.64
TW + SBS2	1.45
TW + SBS3	1.31
TW + SBS1 + PAA^d^^,^ ^e^	1.51
TW + SBS2 + PAA^d^^,^ ^e^	1.33
TW + SBS3 + PAA^d^^,^ ^e^	1.29

a *Contact time: 15 s based on Morris recommendation and current industry practice*.

b *There were eight antimicrobial treatments consisting of: a no treatment Control, tap water (TW), tap water with the addition of either 1, 2, or 3% sodium bisulfate (SBS) indicated as TW+SBS1, TW+SBS2, and TW+SBS3, and the combination of 1, 2, and 3% SBS with 200 ppm of peracetic acid (PAA), represented as TW+SBS1+PAA, TW+SBS2+PAA, and TW+SBS3+PAA*.

c *Mean pH of part dips was determined using a SympHony pH meter (VWR International, Radnor, PA). The mean pH of the solutions prepared for drumsticks was based on the four replicated experiments. There was no pH for the no treatment group (Control) as no solution was prepared*.

d *200 ppm concentration of PAA*.

e *Peracetic acid solution; 39% PAA; Sigma-Aldrich, 3050 Spruce St., St. Louis, MO*.

## Discussion

Although the authors did not evaluate the *Salmonella* recovered in this study for invasion or infectivity, data presented herein is promising as it demonstrates the possibility of SBS to improve food safety. Throughout the course of this study, SBS treatments reduced the concentration of *S*. Enteritidis below the typical infectious dose of ingested *Salmonella* to humans, 10^6^ to 10^8^ CFU, though the infectious dose of *Salmonella* can vary based on the matrix and the immune status of the affected individual (24). Despite the fact that the infectious dose has been reported to be much lower in other studies (25), the log reduction of 1.75 CFU/g on d 3 in the current study demonstrates the ability of SBS to effectively reduce pathogens to a potentially non-infectious dose for those who are not immunocompromised. Therefore, data presented herein warrants further investigations into whether or not treating poultry carcasses with SBS reduces salmonellosis.

The use of PAA has been shown to be an effective antimicrobial in poultry processing and its potential synergism with SBS (25). Two commercial acidifiers, acetic acid and hydrogen peroxide, are individually effective against pathogens. In combining both acids, synergism is demonstrated and yields PAA (26). This effect is likely driven by the acidification of the hydrogen peroxide by acetic acid (26). It is possible that the acidification of PAA may enhance its antimicrobial properties. As SBS is a strong acidifier with a pK_a_ of 1.9 (15), it was important to evaluate the potential synergism between SBS and PAA. As demonstrated by the current study, the combination of 3% SBS and 200 ppm of PAA had a lower pH (1.29) and reduced *S*. Enteritidis more than 1.7 log CFU of *S*. Enteritidis per g of drumstick. Consequently, the combination of SBS with PAA demonstrated similar trends in reducing *Salmonella* as other studies investigating the use of PAA alone. The application of 85 ppm of PAA in a chilling tank resulted in a 1 log reduction (91.8% reduction) of *Salmonella*-positive carcasses (27). In other research investigating the effects of PAA as a post-chill dip (10 or 20 s), Nagel et al. (28) reported a 2-log reduction of *S*. Typhimurium among whole carcasses treated in a 20 s post-chiller dip of 400 or 1000 ppm of PAA. Though the current study indicates the potential combinatorial effect of SBS and PAA, it also confirms the validity of SBS as an antimicrobial when used alone.

In previous studies, the use of SBS alone has demonstrated to be an effective antimicrobial agent. Previously, when SBS was applied as a pre-chill 90 s spray, it resulted in a 2.4 log CFU reduction of *S*. Typhimurium (29). Another study similar to the one conducted herein demonstrated a similar reduction of *Salmonella* was exhibited in research by Yang et al. (30). Yang et al. (30) showed that a 17 s application of 5% SBS in an inside-outside bird wash reduced *Salmonella* by 1.66 log CFU per carcass. As demonstrated in the current and past studies, the acidification of water induced by SBS has the potential to effectively reduce foodborne pathogens, nevertheless, their use may be hindered due to the complex nature of poultry skin.

Although SBS proved to be a potent antimicrobial in the current study, there is a possible buffering effect of poultry meat and skin that may inhibit the competency of antimicrobials on poultry parts. Tan et al. (31) demonstrated that the use of organic acids was capable of reducing *Salmonella* on chicken skin. However, the use of organic acids was only effective after the pH was reduced below 2, with acetic acid being the most efficacious (31). This is consistent as chicken skin exhibits a stronger buffering effect than skin remnants and adipose tissue alone (32). As a result, the efficacy of SBS may be inhibited, but the application of a surfactant in conjunction with SBS may counteract some of the potential buffering ability of poultry skin and meat. This can potentially be overcome with the use of surfactants, which disrupt the surface topography of the skin and reduce the buffering effect. To illustrate the advantage of combining inorganic acids with surfactants, Kim and Day (33) combined hydrogen peroxide, sodium bisulfate, and thymol, a surfactant, and evaluated the effect of the combined solutions on *E. coli* and *S*. Typhimurium. Kim and Day (33) demonstrated the combination yielded a synergistic effect on MIC's as the combination lowered *E. coli* and *S*. Typhimurium three-fold greater than the MIC's of the individual components and reduced both pathogens by 2 logs. As a result, the incorporation of a surfactant such as thymol should be included in future studies to enhance the anti-pathogenic effects of SBS and PAA.

Another factor that may have also played a role in the inhibition of SBS was the acquisition of drumsticks from a local supermarket rather than acquiring them immediately after cut-up in a local processing plant. Therefore, the drumsticks purchased may have had antimicrobials such as PAA already applied to them. Although this may have influenced the results, the effect in the current study would be relatively small as untreated controls were utilized. Thus, any bias toward pretreatment of drumsticks should be accounted for when comparing the results. In addition, because of the small sample size (*n* = 4 per treatment) used in the current experiment, there is a room for future research to validate our current result with a larger sample size.

## Conclusions

The current study demonstrated that there is a greater efficacy on *S*. Enteritidis reduction as SBS concentration is increased, with no visual discoloration and 3% SBS being most effective. Drumsticks treated with 3% SBS, 2% SBS with the addition of 200 ppm of PAA, and 3% SBS with the addition of 200 ppm of PAA had the most significant reductions of *S*. Enteritidis over a 3-d refrigeration period (1.7 log CFU of *S*. Enteritidis per g of drumstick of *S*. Enteritidis). The treatment of drumsticks with 3% SBS demonstrated the effective reduction of *S*. Enteritidis regardless of the presence of 200 ppm of PAA. Therefore, the application of 3% SBS as an antimicrobial part dip has the potential to be an advantageous tool to further reduce the contamination of poultry parts past the post-chilling stages of processing.

Further research should be conducted to determine the effects these specific concentrations of SBS have on the overall shelf life of poultry parts and on diminishing *Salmonella* when combined with a surfactant. In order to determine whether or not efficacy is consistent across all major poultry serovars, SBS needs to be tested with other *Salmonella* serovars. Lastly, studies that optimize the application of SBS to reduce *Salmonella* and determine other potentially synergistic compounds must be conducted. In doing so, investigators will continue to develop potent antimicrobials for poultry processing that will reduce the transmission of pathogens to the food supply.

## Author Contributions

All authors significantly contributed to the work of the current study. DD, JA, KF, and SR designed and prepared the current study with the assistance from CK. DD and JA conducted the experiment. JL analyzed the data and DD wrote the manuscript with assistance from KF, JL, CK, and SR.

### Conflict of Interest Statement

CK is employed by the company Jones-Hamilton. The remaining authors declare that the research was conducted in the absence of any commercial or financial relationships that could be construed as a potential conflict of interest.
